# MicroRNA Regulation of Oxidative Stress-Induced Cellular Senescence

**DOI:** 10.1155/2017/2398696

**Published:** 2017-05-16

**Authors:** Huaije Bu, Sophia Wedel, Maria Cavinato, Pidder Jansen-Dürr

**Affiliations:** Institute for Biomedical Aging Research and Center for Molecular Biosciences Innsbruck (CMBI), Universität Innsbruck, Innsbruck, Austria

## Abstract

Aging is a time-related process of functional deterioration at cellular, tissue, organelle, and organismal level that ultimately brings life to end. Cellular senescence, a state of permanent cell growth arrest in response to cellular stress, is believed to be the driver of the aging process and age-related disorders. The free radical theory of aging, referred to as oxidative stress (OS) theory below, is one of the most studied aging promoting mechanisms. In addition, genetics and epigenetics also play large roles in accelerating and/or delaying the onset of aging and aging-related diseases. Among various epigenetic events, microRNAs (miRNAs) turned out to be important players in controlling OS, aging, and cellular senescence. miRNAs can generate rapid and reversible responses and, therefore, are ideal players for mediating an adaptive response against stress through their capacity to fine-tune gene expression. However, the importance of miRNAs in regulating OS in the context of aging and cellular senescence is largely unknown. The purpose of our article is to highlight recent advancements in the regulatory role of miRNAs in OS-induced cellular senescence.

## 1. Introduction

Cellular senescence, a state of permanent cell growth arrest in response to cellular stress, is characterized by morphological transformations, expression of senescence-associated *β*-galactosidase (SA-*β*-gal), accumulation of the cyclin-dependent kinase (CDK) inhibitor p16^INK4a^, senescence-associated secretory phenotype (SASP), senescence-associated heterochromatin foci (SAHF), telomere shortening, and concomitant-persistent DNA damage response (DDR) [1]. Senescence-inducing stimuli include a variety of intracellular and extracellular stressors. Telomere shortening is found to cause replicative senescence in vitro, although its relevance to human aging remains unclear [2]. Other factors like oncogene activation, tumor suppressor loss, DNA damage, and oxidative stress (OS) are responsible for stress-induced premature cellular senescence [3]. The halting of damaged cells from proliferation has been initially considered as a tumor suppressor mechanism [4], although recent studies showed that the physiological role of senescence also extends to development, tissue repair, inflammation associated with tumor promotion, and aging [1]. Aging is a time-related process of functional deterioration at cellular, tissue, organelle, and organismal level that ultimately brings life to end. Senescent cells accumulate in multiple tissues with age [5], and removal of these cells delays tumorigenesis and age-related disorders in different tissues [6] as well as prolongs lifespan in vivo [7]. It is generally believed that cellular senescence is the driver of the aging process and age-related disorders [3, 8].

The signaling networks and molecular mechanisms underlying the induction and maintenance of cellular senescence are being unraveled. In response to DNA damage and reactive oxygen species (ROS), DDR is activated, in which ATR or ATM kinase blocks cell-cycle progression through stabilization of p53 and transactivation of the CDK inhibitor p21^Waf1/Cip1^ [3]. Other stressors trigger senescence in DDR-dependent or DDR-independent way through activating p19^Arf^, p16^Ink4a^, and pRb [3]. Senescence is a multistep evolving process, in which the cells evolve from early to full senescence [3]. p53/p21 or p16/pRb signaling are important for the early phase of senescence, in which the cells transit from temporal to persistent cell-cycle arrest, whereas extensive changes in chromatin structure and chromosome organization are required in order to progress to full senescence [3, 9]. Alterations in chromatin structure greatly affect the transcriptional program [3], including the upregulation of a group of secreted proteins, such as growth factors, proteases, and proinflammatory cytokines and chemokines, collectively referred to as the senescence-associated secretory phenotype (SASP). SASP is one of the key characteristics that distinguishes senescent cells from quiescent or terminally differentiated cells [1]. Of note, unlike quiescent cells that are metabolically inactive, senescent cells are highly metabolically active. New findings reveal key roles of changes in cell metabolism in the establishment and control of senescent phenotypes [10]. For example, a significant shift to more glycolytic metabolism but also less energetic state was observed as fibroblasts undergo replicative senescence [11]. In line with these findings, mitochondria, the cellular powerhouse, play a key role in pathophysiology of aging, and mitochondrial dysfunction is a major contributor to aging and aging-related diseases [12]. Thus, cellular senescence, as both evolving and static phenotype, is induced and maintained by a complex signaling network, in which genomic surveillance pathways, transcription program, and cellular metabolism are coordinated.

## 2. OS, Cellular Senescence, and Aging

The free radical theory of aging [13] describes one of the most studied mechanisms of aging and aging-related pathologies such as diabetes, cardiovascular diseases, and neurodegenerative diseases. It explains aging at a molecular level by cellular accumulation of oxidative damage to macromolecules, like DNA, proteins, and lipids, and results from failure to maintain antioxidant defenses, mitochondrial function, genomic integrity, metabolic homeostasis, and immune function [14]. ROS are produced from inefficient electron transfer in the mitochondrial respiratory chain and can be enhanced by a number of pathophysiological stimuli like tobacco smoking, UV radiation, and inflammation [15]. In the aging process, increased ROS generation results from mitochondrial dysfunction characterized by accumulation of mitochondrial DNA mutations, impairment of oxidative phosphorylation, impairment of antioxidant defenses [16], and other regulatory inputs. The function of ROS in controlling cellular senescence, aging, and age-associated diseases, as well as its regulation mechanisms, has been recently reviewed [14, 17, 18]. ROS causes senescence associated cell-cycle arrest by triggering DDR pathway to stabilize p53 and transactivate p21 gene expression [19]. In case of persistent DNA damage, p16 is activated via p38/MAPK pathway and nuclear lamin B1 is downregulated, triggering chromatin remodeling coupled with the formation of highly condensed chromatin regions referred to as senescence-associated heterochromatin foci (SAHF) [9]. SASP, the senescence-specific proinflammatory secretome, is closely linked to SAHF and reinforces senescence by coupling with a persistent DDR as a positive feedback loop with the ROS stimulation [20]. Stem cells, a group of self-renewable cells responsible for maintenance of tissue homeostasis and whose dysfunction leads to accelerated aging, age-associated pathologies, or cancer, are also sensitive to ROS level [17]. High levels of ROS impair the function of hematopoietic and neural stem cells, while physiological levels of ROS are required for both their proliferation and their differentiation [17]. Collectively, ROS that trigger aging are referred to as aging-inducing ROS (AIROS) below.

**Figure 1:**
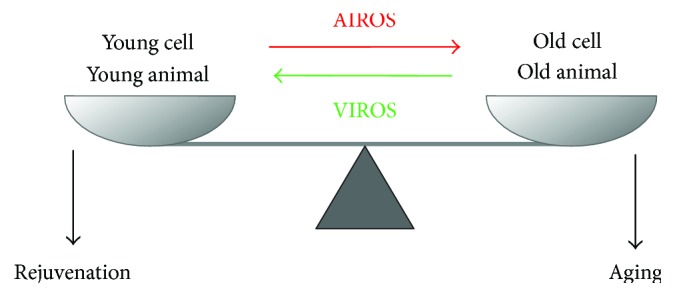
Role of ROS in aging and youthful physiology.

Although widely accepted, the OS theory of aging has been recently challenged due to (1) failure of evidence from increasing antioxidant capacity to prolong lifespan and (2) the fact that several longevity-promoting interventions, for example, caloric restriction or inhibition of target of rapamycin (TOR), in animal models converge by causing activation of mitochondrial oxygen consumption and, sometimes, increased ROS production [21]. In fact, nontoxic levels of ROS, referred to as vitality-associated ROS (VIROS) below, are suggested to promote metabolic health and longevity [21]. An increasing body of evidence shows that besides inducing OS, ROS are able to act as signaling molecules in the maintenance of physiological functions—a process termed redox biology. In contrast to high level of ROS that results in damage to macromolecules, redox biology refers to modulation of ROS levels that activates signaling pathways to initiate biological processes, like proliferation, inflammation, and aging [17]. Depending on ROS concentration, subcellular localization, and species, the cellular response can be either OS-induced damage or redox signaling [17]. For instance, increasing mitochondrial superoxide level extends lifespan, while cytosolic ROS shortens lifespan in *C. elegans* [22]. The function of mitochondrial ROS in increasing immunity to various pathogens suggests a possible link between ROS, immunity, and longevity [23]. In the case of UVB-induced senescence of human diploid fibroblasts (HDFs), ROS is essential for autophagy activation and inhibition of ROS production by antioxidant treatment leads to cell death [24]. The different functions of ROS in aging and senescence are depicted in Figure 1.

In young animals and young cells, youthful physiology and vitality are assured by physiological levels of reactive oxygen species (ROS), referred to as VIROS, which are essential for a plethora of signaling pathways. During aging, increased levels of ROS, probably in combination with an altered spectrum of ROS chemistry and ROS subcellular localization, collectively referred to as AIROS, lead to the accumulation of damage to biological macromolecules contributing to aging.

Taken together, these studies suggest a reorientation of the initial view of ROS as promoter of cellular senescence and aging by causing cumulative damage; instead, ROS act as signaling molecules which are able to elicit either beneficial or detrimental effects depending on intracellular and environmental factors.

## 3. p53, OS, Cellular Senescence, and Aging

p53 plays key and complex roles in cellular response to OS. p53 as a cellular gatekeeper on one side is able to decrease ROS level in order to control OS and repair DNA damage; and on the other side, it can also promote ROS production and induce apoptosis or senescence when the damage is irreversible. In response to physiological OS, p53 reduces intracellular ROS level by inducing antioxidants and regulating metabolism [25]. In this context, antioxidant enzymes including MnSOD, Sestrins, and GP x 1 are involved [25, 26]; other metabolic enzymes like TIGAR, GLS2, and ALDH4 decrease ROS production by either slowing down glycolysis and promoting NAPDH production or strengthening mitochondrial function [25, 26]. In response to high levels of OS, p53 exacerbates OS and executes cellular apoptosis by targeting pro-oxidants including NADPH oxidase, members of pro-oxidant family PIG1–13 (p53-inducible genes 1–13), and proapoptotic molecules BAX and PUMA, as well as inhibiting expression of antioxidants [25, 26].

p53 is also a longevity assurance gene through tumor-suppressing function and a regulator of aging and cellular senescence. In mouse models, altered p53 activity may either suppress longevity and accelerate aging phenotypes or enhance longevity [27–32]. p53 mutant mice with consistent active p53 showed diminished stress tolerance and reduced lifespan [27, 28], while mouse with increased but otherwise normally regulated p53 showed a normal aging [29, 30]. Interestingly, modest and regulated increase of both p53 and Ink4/Arf significantly prolonged longevity and delayed organismal aging [31, 32]. At cellular level, p53 is induced in HDFs when cells are challenged by senescence stimuli, that is, OS, irradiation, or oncogene activation, leading to mitosis skip and subsequent senescence induction [33]. Transient activation of p53 at G2 phase was found to be sufficient for senescence induction [33, 34]. Interestingly, downregulation of p53 by SCF^Fbxo22^ was reported to be crucial for the induction of p16 and SASP [35]. In case of moderate stress challenge, p53 is activated to halt the cell cycle and trigger the repair mechanisms. The reversion of cell cycle arrest after the repair response requires p53 degradation in an ubiquitylation-dependent way [36]. mTOR is identified as a key molecule in determining the outcome of p53 signal to either induce reversible quiescence or irreversible senescence; while maximal activation of p53 blocked mTOR and led to quiescence, partial p53 activation preserved mTOR activity and induced senescence [37]. Other players in decision of cell fates in response to p53 pathway have been reviewed recently [38].

## 4. Changes in Gene Expression during Cellular Senescence

Senescence is a multistep dynamic process with acquired phenotypes. Changes in gene expression profiles have been proposed to be implicated in the process [39]. A time series transcriptome study in replicative senescence revealed changes in the expression of genes related to growth arrest and metabolism during the early onset of senescence and to genes involved in inflammation and immune function-related and growth regulation in the late stage of senescence [39]. Inflammation and the immune function are also found to be common pathways in both replicative and stress-induced premature senescence models [40]. As mentioned previously, in addition to p53/p21 and pRb/p16 signaling, epigenetic modifications including DNA methylation, histone modifications, and chromatin remodeling also play an important role in defining and maintaining the senescence state through regulating gene expression [9, 41]. For instance, both in vivo and in vitro studies show that aging and cellular senescence are associated with genome-wide DNA hypomethylation and focal DNA hypermethylation [42]. Loss of the active chromatin histone marker H3K4me3 at cell-cycle regulatory genes, due to proteolysis, facilitates transcriptional silencing and promotes senescence [43].

## 5. Changes of miRNA Biogenesis during Aging and Cellular Senescence

In the past years, microRNAs (miRNAs) turned out to be important players in controlling aging and cellular senescence [44–46] by regulating gene expression either by translational repression or by mRNA degradation [47]. For example, one of the best characterized aging-associated pathways in *C. elegans*, the IGF signaling pathway, is regulated by lin-4 and its target lin-14. Of note, a global decrease in miRNAs abundance was found in aging of different model organisms, suggesting aging-associated alteration of miRNAs biogenesis [44]. In fact, aging-induced dysregulation of miRNAs biogenesis proteins is reported to promote aging and aging-associated pathologies. Among them, ribonuclease Dicer is most studied and a reduced level was reported in tissues of aged mice and rats, as well as in senescent cells [48–50]. Downregulation of Dicer and multiple miRNAs in adipose tissue is associated with accelerated aging, reduced life span, and stress defense in different model organisms from *C. elegans* to mice and also in humans [48]. A similar phenotype was also observed in primary cerebromicrovascular endothelial cells, where aging-induced downregulation of Dicer1 resulted in altered miRNA profiles and vascular cognitive impairment [50]. Interestingly, longevity-promoting interventions, for example, caloric restriction and metformin supplementation [51], prevent age-related decline of Dicer and miRNA processing and exert Dicer-dependent antisenescence effect [48, 52], while senescence-inducing stimuli, like OS or UV radiation, decrease Dicer expression [48]. Knockdown or knockout of Dicer1 in cells resulted in increased DNA damage and p19Arf-p53 activity [53] and premature senescence [48, 53, 54]. Dicer1 knockout mice showed a shift in metabolism from oxidative phosphorylation to aerobic glycolysis, hypersensitivity to OS, and cell senescence phenotype in vivo [48, 53, 55]. In comparison to Dicer, the role of proteins of microprocessor complex in cellular senescence is less studied. Although several studies pointed to an antisenescence role of DGCR8, the function of Drosha remains to be unraveled [56–60]. Loss of miRNA synthesis through blocking DGCR8 expression in adult *C. elegans* showed accelerated aging and reduced lifespan [57]. Knockdown of DGCR8 triggered a dramatic antiproliferative response and consequently senescence phenotype characterized by upregulation of p21 in primary fibroblasts of human and mouse origin [58]. In addition, several senescence regulating proteins are reported to regulate DGCR8. Among them, p63, an essential transcription factor for epidermal function and an antiaging molecule, directly promotes the transcription of DGCR8 and Dicer [60]; ING proteins (inhibitor of growth), a family of tumor suppressors and senescence promoters, repress DGCR8 expression [56, 59]. Drosha mRNA was reported to be moderately reduced in adipose tissue of aged mice [48] and the protein level was downregulated in replicative senescent WI-38 fibroblasts [61]. Although knockdown of Drosha in IMR90 human primary fibroblasts showed an antiproliferative effect [58], its downregulation in WI-38 fibroblast, however, revealed no effects on cellular senescence [61]. Further efforts are needed to clarify the role of Drosha in aging and cellular senescence.

## 6. miRNA Stability in Cellular Senescence

Although the mechanisms of miRNA biogenesis have been intensively investigated since the last years, processes regulating miRNA stability remain to be explored. miRNAs have been generally considered as stable molecules with half-life of days long [62, 63], while some miRNAs, for example, brain-enriched miRNA-9, miRNA-125b, are actually short lived with half-life of no more than few hours [64]. It is now clear that the absolute levels of mature miRNAs are also controlled by *cis*- and *trans*-acting factors that directly affect stability [65]. Under them, the association of mature miRNAs with Argonaute (AGO) is critical for miRNA stability [66], probably due to protection from degradation by ribonuclease. GW182, a downstream effector of AGO and an AGO-interacting partner essential for gene silencing is also important for stability of miRNAs [67]. In addition, the stability or degradation of mature miRNAs seems to be miRNA and modification specific. For example, 3′ adenylation of specific miRNAs like miR-122, miR-145, and Let-7d by PAPD4, a noncannonical poly(A) RNA polymerase, stabilized them [68], while PAPD5-mediated 3′ adenylation of miR-21 leads to its degradation [69]. The exonuclease XRN-2 also shows selectivity on degradation of its miRNAs substrates [70, 71]. Further efforts are needed to elucidate the mechanisms of altered miRNA stability in the context of miRNA modification and selectivity of miRNA stabilization and/or degradation enzymes.

If miRNA stability changes during cellular senescence is so far, to our knowledge, not known. In *Drosophila*, 2′-O-methylation of miRNAs, a modification occurs on nearly all small RNAs in plants to protect them from degradation [72, 73], was reported to be increased in aged animals [74]. An increased loading of these miRNAs to AGO2 and stabilizing was found to underlie the age-associated alteration and lack of 2′-O-methylation of miRNAs results in accelerated neurodegeneration and shorter life span [74]. Further researches on miRNA stability and degradation mechanisms in cellular senescence and aging are needed to identify its impact on age-associated process and may provide potential new targets to interfere the process.

## 7. miRNA Biomarkers of Senescence and Aging

A number of miRNAs, including miR-106b, miR-125b, miR-126, miR-146a, miR-21, miR-22, miR-29, miR-210, miR-34a, miR-449a, miR-494, and miR-17-92 cluster and miR-200 family, have been found to be differentially expressed in senescent cells or aged tissues and play a role in cellular senescence [75–77]. Recently, miRNAs have been found extracellularly and function in intercellular communication upon taken up by recipient cells [78]. The fact that circulating miRNAs are packed in the form of microvesicles, proteins including AGO2 or lipoprotein protects them from degradation [79]. The stability of miRNAs in the circulation and in body fluids, their tissue and disease specificity, and the easy and reliable quantification methods make them feasible as potential biomarkers [78]. Several miRNAs including miR-126, miR-130a, miR-142, miR-21, and miR-93 detected in blood samples have been found in several studies to be associated with human aging [80]. Furthermore, circulating miRNAs can also help to define healthy aging by comparing nonagenarians, centenarians and their offspring to patients with age-related diseases [80]. For example, miR-21 level is increased with aging process, aging associated diseases, and cancers but decreased in subjects older than 80 years and in centenarians, implying that low levels of miR-21 are beneficial for longevity and could be a promising biomarker candidate of healthy aging [80]. A miRNA profiling of serum samples collected longitudinally at the ages of 50, 55, and 60 from individuals with documented lifespans was recently analyzed [81]. When the participants were subdivided into long-lived and short-lived subgroups, a multiplex of aging biomarker with the expression of miR-211-5p, miR-374a-5p, miR-340-3p, miR-376c-3p, miR-5095, and miR-1225-3p was proposed to be significantly differentially expressed and correlated with lifespan, providing a basis for investigating miRNAs as potential longevity predictors and regulators of aging process [81]. Circulating miRNAs from blood samples have been also investigated as biomarkers of aging-associated diseases, like cardiovascular diseases, neurodegenerative diseases, type 2 diabetes mellitus, and bone diseases [78, 82, 83]. Body fluids other than blood samples are also a reservoir of miRNA biomarkers. For example, a pilot study on exosomal miRNAs in saliva suggests that miR-24 may be potential biomarkers for aging [84]. The levels of miR-34a, miR-125b, and miR-146a in cerebrospinal fluid of patients with Alzheimer's disease were lower compared with those of control subjects, implying a possibility to use miRNAs detected in cerebrospinal fluid as biomarkers for neurodegenerative diseases [85]. Further efforts are needed to identify consensus miRNA biomarkers not only as indicators of aging process and aging-associated disease but also as longevity predictors and eventually therapeutic approaches to modulate the aging process.

## 8. miRNAs Regulating OS in Cellular Senescence

**Figure 2:**
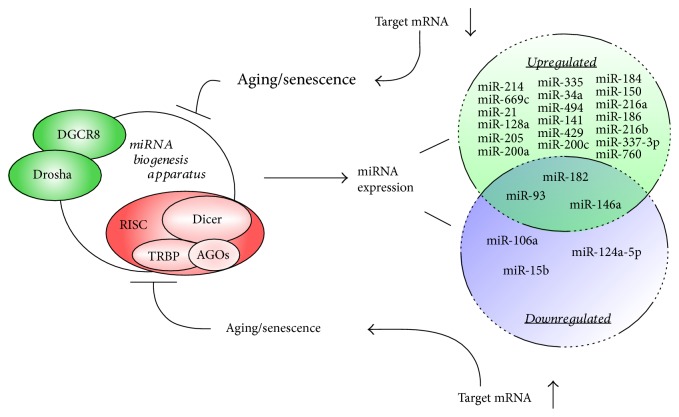
Role and regulation of micro-RNAs in aging and senescence.

**Figure 3:**
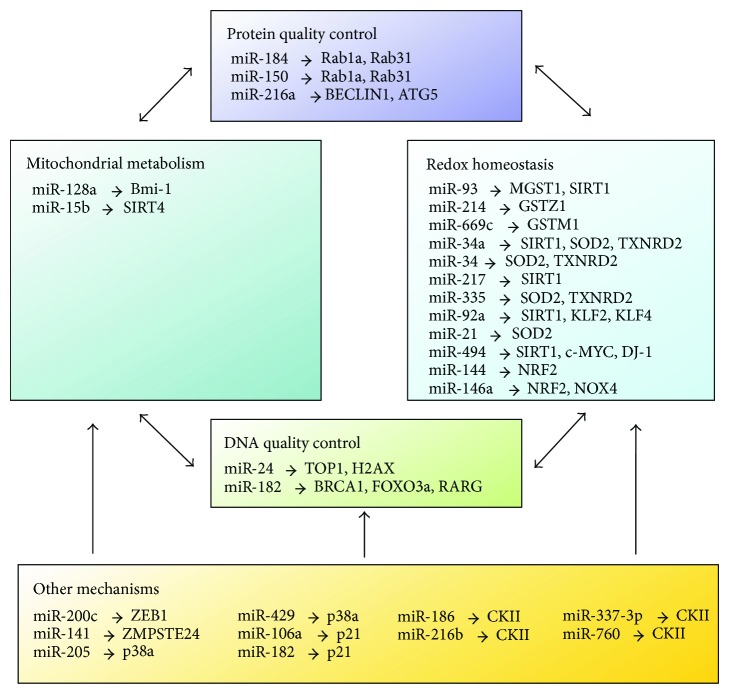
MicroRNAs and their mRNA targets as modulators of redox biology, mitochondrial metabolism, and quality control of DNA and proteins. The maintenance of DNA and protein quality is crucial for the preservation of youthful physiology in animals. Accordingly, mechanisms of DNA and protein quality control (QC) were identified as key targets for cellular senescence and aging. The performance of both QC mechanisms is affected by both mitochondrial and cytosolic ROS. Depicted here are known functions of microRNAs as mediators between ROS production and QC mechanisms. The final outcome of this regulatory circuit is further modulated by other (additional) mechanisms which are currently incompletely understood.

OS can regulate miRNA-mediated gene silencing in senescence induction, by either affecting the miRNA producing organelle (Figure 2) or regulating (upregulation or downregulation) the expression of certain specific miRNAs (see below, Figure 3).

The function of miRNA biosynthesis apparatus is compromised during organismic aging and in cellular senescence, leading to a general decline of microRNA availability with age. On the other hand, cellular pathways regulating OS are fine-tuned by specific microRNAs (up- or downregulated) which alter the expression of cellular target genes with the final result to induce and/or enforce senescence and aging.

In a study of RAS-induced premature senescence of fibroblasts, AGO2 was found to be a substrate of protein tyrosine phosphatase 1B (PTP1B), a major target of RAS-induced ROS [86]. Inactivation of PTP1B and consequently phosphorylation of AGO2, which inhibits loading and function of miRNAs, were necessary and sufficient for RAS-induced senescence, implying the importance of OS-mediated inhibition of miRNA function in senescence induction [86]. On the other hand, miRNAs can generate rapid and reversible responses and, therefore, are ideal players for mediating adaptive responses against stress through their capacity to fine-tune gene expression [87]. In the following section, we will summarize and categorize the miRNAs that regulate OS in cellular senescence according to their targets (Figure 3).

### 8.1. Redox Homeostasis

miR-93, miR-214, and miR-669c were found to be overexpressed in aged mouse livers [88]. Through proteomics approach, glutathione S-transferases, such as microsomal glutathione S-transferase 1 (MGST1), glutathione S-transferase zeta 1 (GSTZ1), glutathione S-transferase mu 1 (GSTM1), and glutathione S-transferase theta-1 (GSTT1), were identified as targets of these miRNAs, implying the decline of oxidative defense mechanisms through miRNAs in aging liver [88]; miR-93 was also reported to be upregulated in aged rat liver where Sirtuin1 (SIRT1), in addition to MGST1, was identified as its target [89]. SIRT1 is a nicotinamide adenine dinucleotide- (NAD^+^-) dependent deacetylase that regulates crucial cellular functions and is associated with aging and longevity. It mediates stress resistance and its expression levels decline in aging organisms, where OS occurs [90]. A variety of miRNAs regulate SIRT1 expression [90]. Of note, miR-34a was found to induce cellular senescence by targeting SIRT1 in different tissues [89, 91–93]. miR-217 was reported to induce a premature senescence-like phenotype and impaired angiogenesis in endothelial cells through directly targeting SIRT1 and modulating its deacetylase activity [94]. miR-92a was found to exacerbate endothelial dysfunction in response to OS through targeting SIRT1, Krüppel-like factor 2, and Krüppel-like factor 4, which are key molecules in endothelial cell homeostasis [95].

miR-34a was also reported to target genes in the antioxidant pathway other than SIRT1 to contribute to OS-mediated cellular senescence. Together with miR-335, miR-34a was found to be upregulated in aged rat kidney having superoxide dismutase 2 (SOD2) and thioredoxin reductase 2 (TXNRD2) as their targets, respectively [96]. Overexpression of miR-335 and miR-34a induced premature senescence of young mesangial cells via suppression of both antioxidative enzymes with a concomitant increase in ROS [96]. SOD2 is also a target of miR-21 in human angiogenic progenitor cells (APCs). Reversed correlation of miR-21 and SOD expression was seen in APCs from patients with coronary artery disease. Upregulation of this miRNA increased cellular ROS generation and impaired migratory capacity of APCs [97].

miR-494 was observed to be overexpressed in both replicative senescence and OS-induced premature senescence of HDFs. Its overexpression also promoted a senescent phenotype, as well as enhanced DNA damage and intracellular ROS generation [98]. In line with this, several OS regulators were found to be miR-494 targets. For instance, both c-MYC and SIRT1 were identified as direct targets in pancreatic cancer cells and tissues, where miR-494 is often found to be downregulated. Overexpression of miR-494 could inhibit proliferation of pancreatic cancer cells through induction of apoptosis, G1-phase arrest, and senescence [99]. DJ-1, an oxidative sensor and molecular chaperone, whose dysfunction contributes to Parkinson's disease, was also found to be posttranslationally repressed by miR-494. This miRNA reversely correlated with DJ-1 level in vivo and rendered cells more susceptible to OS, resulting in loss of dopaminergic neurons in mice [100].

Caloric restriction (CR) is a well-accepted lifespan prolongation intervention method described for most model organisms from worms to primates. In a study conducted to elucidate the mechanism protective of moderate CR on cerebrovascular cells of aged rats, age-related increase in OS was found to be positively correlated with miR-144 and negatively correlated with miRNA target transcription factor of antioxidant gene *Nrf2*. CR significantly decreased miR-144 levels and restored NRF2 expression, indicating that CR confers antioxidative effects through regulating miR-144-NRF2 axis [101]. Actually, as master regulator of redox biology through induction of antioxidant defense, NRF2 is targeted by various miRNAs in order to “fine-tune” the redox homeostasis, and these miRNAs have been reviewed elsewhere [102]. In a recent miRNA profiling study addressing age-related changes in NRF2 protein homeostasis using rat hepatocytes, miR-146a was found to be significantly upregulated in aged liver and identified as a new NRF2-targeting miRNA [103]. Interestingly, the effect of miR-146a in modulating redox biology seems to be tissue dependent. During replicative senescence of human endothelial cells, this miRNA is continuously decreased in higher passage cells and its overexpression was found to delay the appearance of the senescence-like phenotype through direct targeting of NADPH oxidase 4 (NOX4) protein, a major ROS generator in HUVEC cells [104, 105].

### 8.2. DNA Quality Control

miR-24 was previously found by us to exacerbate OS induced cellular senescence through directly targeting DNA topoisomerase I (TOP1) [106]. Since TOP1 plays a role in genomic stability [107], we suggested that in the OS-induced premature senescence model of HDFs, TOP1 attenuates cellular senescence potentially due to its physiological function to maintain genomic stability. miR-24 was also reported by others to target H2AX, a key double-strand break repair protein, in terminally differentiated hematopoietic cell lines and to render cells hypersensitive to DNA damage [108]. In highly differentiated CD8+ T cells, miR-24 was found to be upregulated upon etoposide treatment and to sensitize them to apoptotic cell death [109].

Excess intracellular ROS in normal human fallopian tube epithelial cells upregulates miR-182. miR-182 overexpression triggers significant cellular senescence in a p53/p21-dependant way. However, in cells with p53 mutations, miR-182 overexpression no longer increases p21 expression but functions as an “Onco-miR” to enhance senescence bypass in cells exposed to OS or DNA damage in vitro and in vivo [110]. Since miR-182 is reported to target DNA repair genes, BRCA1 and FOXO3a [111], a role of slowing down DNA damage repair by miR-182 in cells with dysfunctional p53 is suggested underlying miR-182-mediated tumorigenesis [110].

### 8.3. Protein Quality Control

Major cellular mechanisms controlling protein quality control during aging are autophagy and the 20S proteasome. Autophagy activation has been considered as part of the cellular responses to excessive OS, eliminating unwanted, damaged, or oxidative structures, thus favoring antiaging mechanism [112]. Alteration of the cellular quality control mechanism contribute to aging and aging-associated pathology [113]. miR-184 and miR-150 were found to be upregulated in aged rat kidney. Autophagy-associated proteins Rab1a and Rab31 were identified as targets of both miRNAs. Transfection of kidney cells with mimics of miR-184 and miR-150 suppressed the expression of both proteins and consequently lowered autophagy activity, increasing cellular oxidative products that lead to cellular senescence [114]. miR-216a is induced during endothelial aging and is reported to repress oxidized low-density lipoprotein- (ox-LDL-) induced autophagy. Two autophagy-related genes, Beclin1 (BECN1) and ATG5 are putative targets of miR-216a, whose expression reversely correlated with putative targets in vitro and in vivo. BECN1 was identified as a direct target of miR-216a, implying that miR-216a controls OS induced autophagy in HUVECs by regulating intracellular levels of BECN1 and may have a relevant role in aging-associated cardiovascular disorders [115].

### 8.4. Mitochondrial Metabolism

In a search of a tumor-suppressive role of miRNAs in medulloblastoma, miR-128a, which is downregulated in this type of tumor, was found to inhibit cancer cell growth by promoting senescence, through increased ROS production, upregulation of p16 protein level, and increased number of SA-*β*-Gal positive cells [116]. Bmi-1 oncogene, which is important for mitochondrial function and redox homeostasis [117], was identified as a direct target of miR-128a, indicating novel regulation of ROS by miR-128a via the specific inhibition of the Bmi-1 oncogene [116]. SIRT4, a member of sirtuin family exclusively localized in mitochondria, plays an important role in mitochondrial metabolism [118]. Upregulation of SIRT4 was found to be inversely associated with miR-15b in replicative, stress-induced cellular senescence and photoaged human skin in vivo. SIRT4 was identified as a direct target of miR-15b, whose inhibition promotes mitochondrial ROS generation and mitochondrial dysfunction in a SIRT4-dependent manner, linking the miR-15b-SIRT4 axis to senescence-associated mitochondrial dysfunction [119]. In addition, miR-15 was also found to be downregulated in UVB-induced senescence of human dermal fibroblasts [120].

### 8.5. Other Mechanisms

miR-200 family members are often reported to be associated with cellular senescence. For instance, miR-141 is overexpressed in replicative and HDAC-inhibitor-induced senescence of human mesenchymal stem cells [121]. ZMPSTE24, an enzyme involved in the posttranslational maturation of lamin A and responsible for the prelamin A accumulation related to cellular senescence, is found to be a direct target of miR-141 [121]. miR-200 family members, especially miR-200c and miR-141, are upregulated in OS-induced HUVEC cells [122]. Similar to hydrogen peroxide treatment, miR-200c overexpression also induced growth arrest, apoptosis, and senescence in HUVEC cells. E-cadherin transcriptional repressor zinc finger E-box-binding homeobox 1 (ZEB1), a molecule downregulated by OS, was identified as a target of miR-200c. Downregulation of ZEB1 was required for miR-200c-mediated effects [122]. miR-200 family members have been previously reported to target p38*α*, modulate OS response and chemosensitize cancer cells [123]. A similar mechanism was proposed in a ROS-dependent chemotherapeutic drug-induced senescent model of HDFs, where metformin was found to sensitize the doxorubicin-treated cells to senescence through upregulation of miR-205, together with miR-200 family members miR-200a, miR-141, and miR-429 [124].

In a screen of OS-induced premature senescence in primary cultures of HDFs and human trabecular meshwork (HTM) cells, miR-106a was found to be downregulated in both models and p21 was identified as its target [125]. miR-106a was also downregulated in several human models of cellular aging in vitro and in vivo [46]. Among the upregulated miRNAs, miR-182 was found to target retinoic acid receptor gamma (RARG), whose level is downregulated in both senescent models [125]. The function of RARG in cellular senescence requires more study. Protein kinase CKII is a ubiquitous serine/threonine kinase that catalyzes the phosphorylation of a large number of cytoplasmic and nuclear proteins. Its downregulation is found to induce cellular senescence in both HDFs [126] and human colon cancer cells in p53/p21-dependent way [127]. In a search of CKII targeting miRNAs, miR-186, miR-216b, miR-337-3p, and miR-760 were identified. Overexpression of these miRNAs altogether increased cellular senescence and ROS production, which could be antagonized by CKII overexpression [128]. miR-125a-5p was downregulated in human brain microvessel endothelial cells by ox-LDL, a risk factor for vascular diseases by inducing proinflammatory and proatherogenic responses [129]. Overexpression of this miRNA increased nitric oxide production and decreased ROS production, consequently reducing senescence and apoptosis. miR-125a-5p also improved endothelial cell function and decreased cell adhesion to leukocytes, indicating an anti-inflammatory role by decreasing leukocyte recruitment [129].

## 9. Conclusions

Although a relatively new field of research, miRNAs add substantial complexity to the regulation of aging processes and cellular senescence. On one side, a single miRNA can regulate the expression of hundreds of genes from different signaling pathways, which means the whole signaling network could be reset by modulating the expression of one single miRNA. In contrast, miRNAs as players of adaptive stress response could act both as promoters and inhibitors of senescence, depending on the type of stress, the cell or tissue where they are located, and the molecular context in which they play a role. Further efforts are needed to explore the modulatory role of additional miRNAs in OS- and stress-induced cellular senescence. Especially important is to distinguish the function of specific miRNAs in specific cell or tissue types, where the knowledge gained on cell culture level will be applied to the organismal level.
